# Basal Plane Bending of Homoepitaxial MPCVD Single-Crystal Diamond

**DOI:** 10.3390/ma13204510

**Published:** 2020-10-12

**Authors:** Xiaotong Han, Peng Duan, Yan Peng, Xiwei Wang, Xuejian Xie, Jinying Yu, Xiufei Hu, Dufu Wang, Xiaobo Hu, Xiangang Xu

**Affiliations:** 1 State Key Laboratory of Crystal Materials, Institute of Novel Semiconductors, Shandong University, Jinan 250100, China; hanxiaotong698@163.com (X.H.); 13256998266@163.com (P.D.); xiexj@sdu.edu.cn (X.X.); 15774609606@163.com (J.Y.); huxiufei0812@163.com (X.H.); xbhu@sdu.edu.cn (X.H.); xxu@sdu.edu.cn (X.X.); 2 Jinan Diamond Technology Co., Ltd., Jinan 250100, China; wangdufu@163.com

**Keywords:** basal plane bending, homoepitaxial single-crystal diamond (SCD), high-resolution X-ray diffraction, growth temperature

## Abstract

We report herein high-resolution X-ray diffraction measurements of basal plane bending of homoepitaxial single-crystal diamond (SCD). We define SCD (100) as the base plane. The results revealed that growth parameters such as temperature, growth time, and basal plane bending of the substrate all affect the basal plane bending of SCD. First, the basal plane bending of SCD depends mainly on the substrate and becomes severe with increasing basal plane bending of the substrate. The SCD growth experiments show that the basal plane bending increases with elevated growth temperature and increased growth time. Finally, to understand the mechanism, we investigated the substrate-surface temperature distribution as a function of basal plane bending of SCD fabricated by chemical vapor deposition (CVD). This allowed us to propose a model and understand the origin of basal plane bending. The results indicate that an uneven temperature distribution on the substrate surface is the main cause of the base-plane bending of CVD diamond.

## 1. Introduction

Single-crystal diamond (SCD) has a wide band gap, high thermal conductivity, high breakdown voltage, high carrier mobility, and strong resistance to radiation, which earns it the name “the ultimate semiconductor” [1,2,3,4,5,6]. It can be used in deep ultraviolet detectors, particle detectors, high-power, high-voltage. and high-frequency electronics, etc. [7,8]. Motivated by these promising applications, the growth of large-size, high-quality SCD by high pressure high temperature (HTHP) and microwave plasma chemical vapor deposition (MPCVD) has become an important research topic [9,10].

The MPCVD technique has now led to the fabrication of 2-inch mosaic SCDs. In parallel with the improvement on SCD size, improving the quality assessment of SCDs becomes important for their use as semiconductor devices. In general, basal plane bending in SCDs degrades the crystal quality by inducing dislocations and even cracks [11,12,13,14]. In addition, the relationship between basal plane bending and growth parameters has been studied. Hock et al. [15] investigated in situ lattice plane bending during crystal growth and demonstrated that lattice-plane bending and thermo-elastic stress vary with growth rate. Yang et al. found that substrate attachment strongly affects basal plane bending and plastic-deformation-induced dislocations [11]. Sumathi [16] used X-ray diffraction to obtain a basal plane bending of 100 arcsec in AlN, which suggests a high structural homogeneity in the crystals. However, no reports yet exist on basal plane bending in SCDs.

This work thus presents a detailed X-ray diffraction study of the basal plane bending in homoepitaxial SCD deposited by MPCVD. The results indicated that spatial variations of basal plane bending are approximately spherical. In addition, we discuss how the growth temperature, the substrate quality and the growth time affected the bending radius. Furthermore, we propose a mechanism whereby the temperature gradient affects basal plane bending in SCD.

## 2. Materials and Methods 

Basal plane bending was measured by using a Bruker D8 Discover high-resolution X-ray diffractometer (HRXRD) (D8 Discover, Bruker, Germany) operating with Cu Kα1 radiation at 40 kV with a current of 40 mA. The X-ray beam was scanned in 1 mm steps from one edge of the substrate to the other.

**Figure 1:**
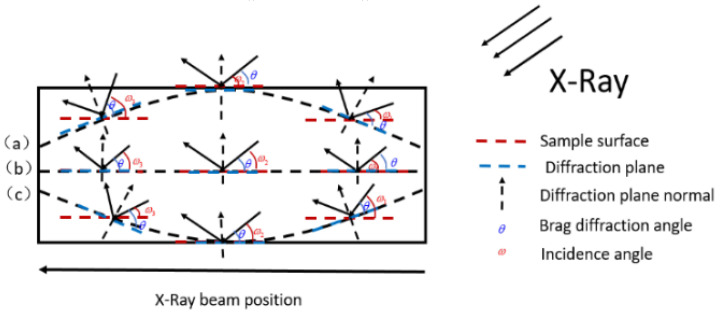
Schematic diagram of HRXRD measurement of basal plane bending. Panels (**a**)–(**c**) show that the basal planes are convex, flat, and concave, respectively.

**Figure 2:**
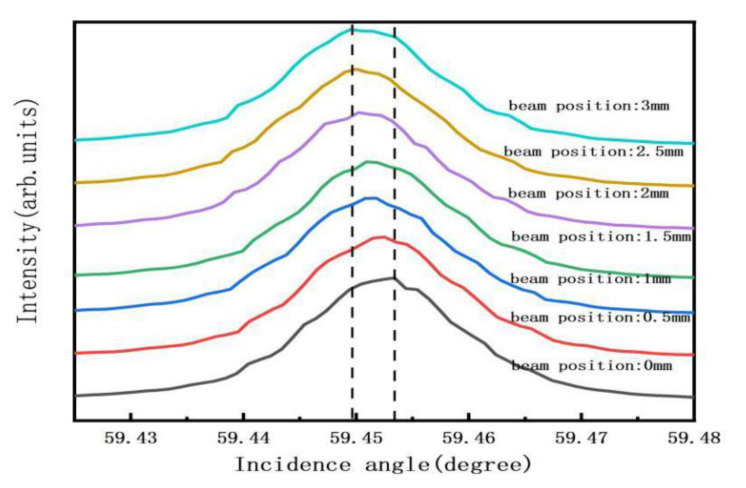
(400) rocking curves at different positions by HRXRD measurement.

We applied the basic principle of X-ray diffraction to detect basal plane bending [17,18]. When the basal plane is flat, the normal of the diffraction plane remains the same regardless of the beam positions. As a result, the angle of incidence is independent of the beam position (*ω*1 = *ω*2 = *ω*3; where *ω* is the angle between the incident beam and the sample surface), as shown in Figure 1b. However, when the basal plane is bent, the normal orientation of the diffraction plane will vary with the beam positions. For a convex (concave) basal plane, as shown in Figure 1a,c, the angle of incidence increases (decreases) monotonically as the X-ray beam is scanned over the sample surface, so *ω*1 < *ω*2 < *ω*3 (*ω*1 > *ω*2 > *ω*3). Figure 2 depicts the variation of relative ω(400) rocking curve peak position along with the beam position across one side to another side of the diamond plate. Upon scanning in a given direction from one edge of the wafer to the other, *ω* gradually changes and the bent basal plane forms an approximately spherical surface. Using the slope of *ω* as a function of scanning distance *x*, *ω* gradually changes and the bent basal plane forms an approximately spherical surface. From the slope of *ω* as a function of scanning distance *x*, the radius *R* of the bending plane is given by

(1) $$R={\left(\frac{d\omega }{dx}\right)}^{-1}$$

where *R* [19] is the radius of the basal plane in the plane of diffraction and *ω* is measured in radians. 

The MPCVD used in our studies is ARDIS-300 made by Optosystems Ltd. (Moscow, Russia) equipped with a 2.45 GHz/6 kW microwave reactor. A detailed description of the chamber geometry and the deposition reaction system is given by Wang [20]. A double interference infrared radiation thermo pyrometer is installed to measure the temperature of the substrate with an emissivity of 0.1 through a slit of 2 mm. The substrate surface temperature was maintained by self-adjusting the microwave power input through feedback from the thermo pyrometer. We measured the temperature distribution by moving the thermometer installed on a three-dimensional displacement platform.

Three groups of experiments were carried out. Table 1, Table 2 and Table 3 summarize the growth conditions. To compare how the substrate affects basal plane bending, we used (100)-oriented HTHP and MPCVD SCD as substrates for sample H and M. The substrates were purchased from Jinan Zhongwu New Material Co. Ltd. (Jinan, Shandong, China). For SCD growth, the concentration ratio of methane to hydrogen was 3%, the pressure was 275 torr and the growth time was 4 h. The samples labelled H1–H4 were grown separately at 900, 1000, 1100, and 1150 °C, respectively. Table 2 details the growth conditions. Table 3 is for sample H3, which was grown in three cycles. After each growth period of 4 h, HRXRD was used to evaluate how growth time affects basal plane bending.

## 3. Results and Discussion

**Figure 3:**
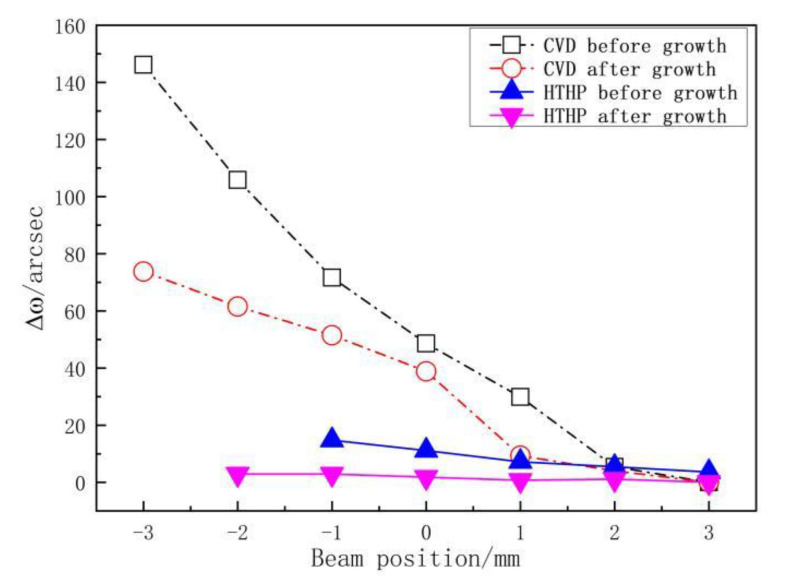
Peak position of *ω*(Δ*ω*400) as a function of beam positions on the diamond plane.

For sample H1 and M1, the basal plane bending was measured before MPCVD growth and after growth. Figure 3 shows the relative *ω* (Δ*ω*400) peak positions as a function of beam positions. As the beam moves from one edge of the substrate to the other, the relative *ω* (Δ*ω*400) peak positions decrease linearly, which indicates spherical bending of the basal plane. The shifts in peak position before and after the MPCVD growth of the H1 sample were 14.76 arcsec and 2.88 arcsec and the corresponding radius of the basal plane bending 74.05 and 358.17 m, respectively.

The peak positions of sample M1 before and after growth were 146.16 arcsec and 73.80 arcsec, respectively, which correspond to basal plane curvature radius of 8.47 and 16.78 m. The results show that (1) the curvature radius of SCD after MPCVD growth is closely related to the substrate curvature, (2) the curvature radius of SCD grown on a HTHP substrate is much greater than that of SCD grown on a MPCVD substrate. The plane bending of SCD is inherited from the substrate, which indicates that the substrate plays an important role in determining the SCD basal plane bending. This mechanism is similar to dislocations inheredity which can propagate from the substrate to the epilayer. To reduce the basal plane bending thus requires the use of high-quality substrates without basal plane bending.

**Figure 4:**
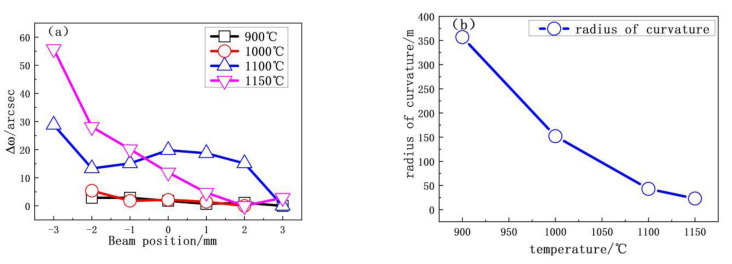
(**a**) Peak position of *ω*(Δ*ω*400) as a function of beam position at several different temperatures. (**b**) Radius of curvature as a function of the growth temperatures.

For SCD grown on the HTHP substrate, the basal plane bending radius exceeds 50 m. We investigated how growth temperature and growth time affected the basal plane bending. Figure 4a shows relative ω(Δω400) peak positions as a function of beam position for samples H1 to H4. The slope of the curve increases with growth temperature, which translates into a reduced radius of curvature of the SCD base plane. As shown in Figure 4b, the radius of the SCD basal plane curvature decreases monotonically with increasing growth temperature. At a growth temperature of 1150 °C, the radius of the basal plane curvature reaches its minimum of 23.40 m.

**Figure 5:**
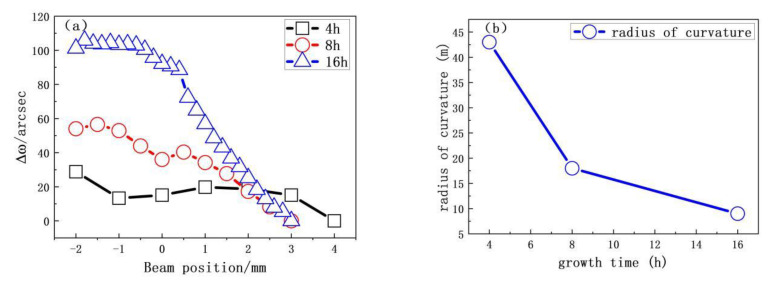
(**a**) Relative position *ω*(Δ*ω*400) as a function of X-ray beam position on the diamond plane for different growth times. (**b**) Radius of curvature as a function of the growth time.

For sample H3, the relative ω(Δω400) peak positions do not decrease linearly. However, with increasing growth time at a given growth temperature, the slope of the curve gradually increases (see Figure 5a). As shown in Figure 5b, the radius of basal plane curvature is 10.20 m for a total growth time of 16 h. Thus, longer growth time leads to more severe basal plane bending.

These results all indicate that the basal plane bending of SCD is affected by temperature, growth time, and substrate. In addition, the basal plane bending is related to the temperature gradient and the substrate attachment. Ha et al. [21] used the finite-element method ANSYS software (Swanson Analysis Systems, Inc, Canonsburg, PA, USA) to simulate the stress distribution during the growth of free and solid seeds and proposed that negative shear stress generates base dislocations in the crystal. By conducting a symmetric Laue geometry transmission test on a primary single crystal containing nitrogen-doped stripes obtained under different growth conditions, Seitz et al. [17] found that the basal plane bending of the single-crystal front end was consistent with the isotherm of the single crystal. In MPCVD growth, the SCD substrate uses open backside attachment, so substrate attachment is not the source of the bending. Based on the model proposed by Harris and Goodwin [22,23,24]. Silva et al. [25] simulated the temperature distribution in the SCD growth chamber and found a large temperature difference between the center and the edges of the chamber. We thus consider herein the effects of temperature gradients.

**Figure 6:**
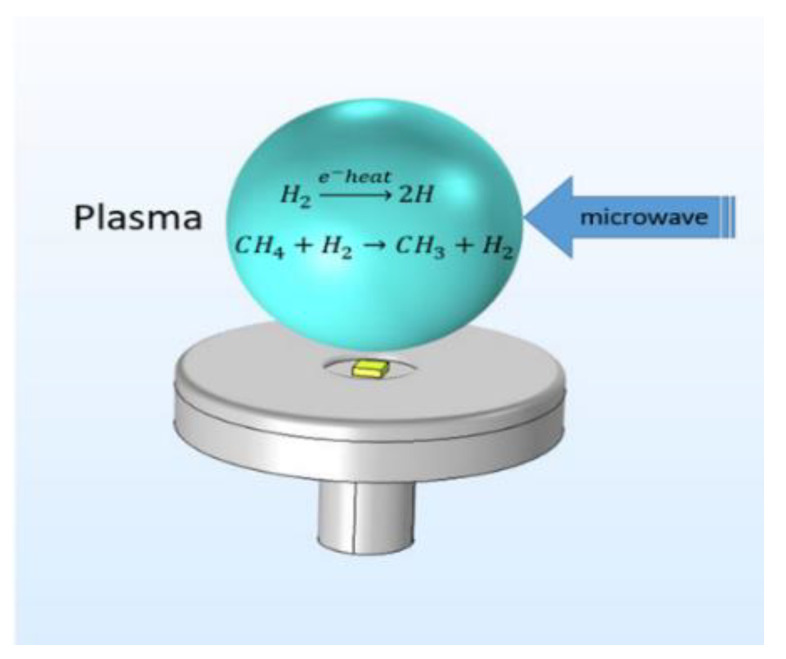
Schematic diagram of synthetic diamond grown by plasma chemical vapor deposition (MPCVD).

In MPCVD, the substrate is heated by the thermal radiation and conduction of the plasma ball to reach a suitable deposition temperature (see Figure 6).

Therefore, the deposition temperature of the substrate surface depends on the distance from the surface of the substrate crystal to the plasma. Because the plasma is spherical, the center of the plasma ball is closer to the surface of the substrate crystal; that is, the temperature of the surface of the substrate crystal is highest near the center of the plasma ball and lower near the periphery of the plasma ball.

**Figure 7:**
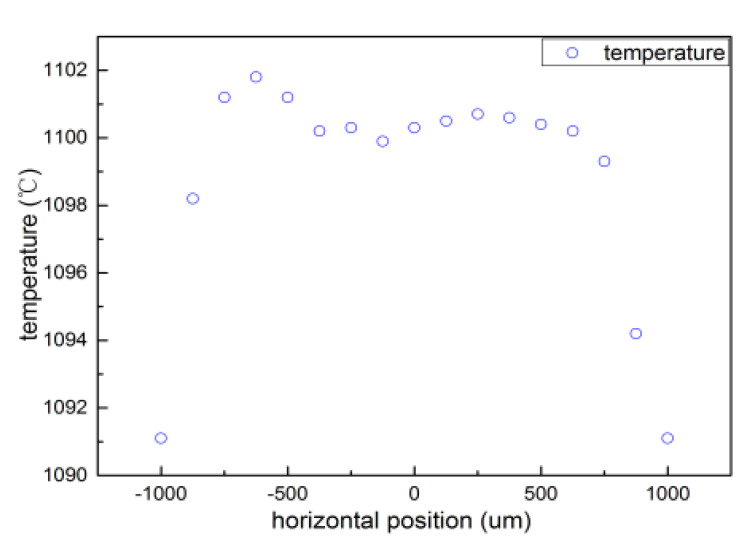
Distribution of sample surface temperature at 1100 °C.

There are two ways to change the temperature gradient of the substrate surface: One way is to change the growth temperature of the substrate surface, and the other way is to change the distance between diamond and plasma. Figure 7 shows the surface temperature distribution over the substrate. The temperature gradient on the surface was 40 °C/mm at 1100 °C. Wang [20] reports that the temperature gradient increases with increasing growth temperature, which in turn increases the thermal stress, causing the SCD basal plane to bend. Higher temperatures lead to more severe base plane bending.

**Figure 8:**
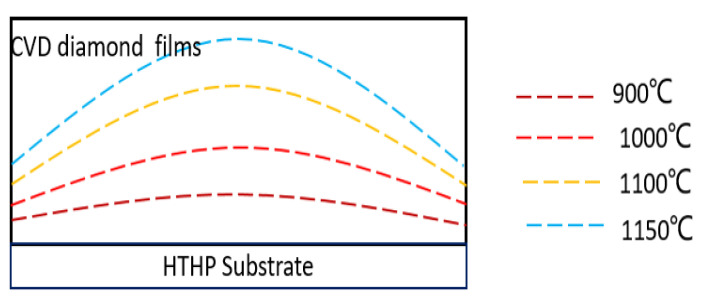
Results of proposed basal plane bending model for different growth temperatures.

Figure 8 illustrates the bending model of the basal plane at different growth temperatures. The substrate holders could also play an important role in the deposition of SCD [26,27].

**Figure 9:**
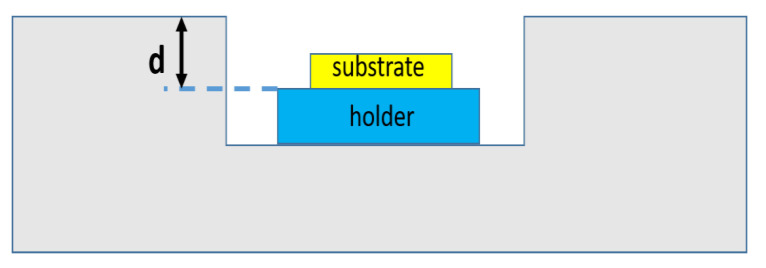
Pocket holder design for single-crystal diamond (SCD) synthesis.

**Figure 10:**
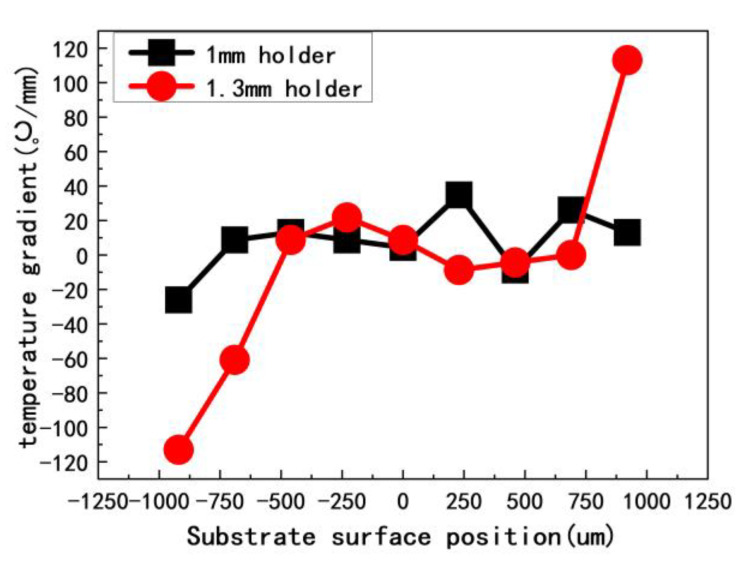
Temperature gradient as function of substrate surface positions for different holder heights.

Figure 9 shows the pocket holder design. In this design, the holder height d significantly affects the temperature gradient of the sample surface. Figure 10 shows the temperature gradient as a function of position on the substrate surface and for different holder heights. The results show that, for d = 1.3 mm, the temperature gradient at the surface of the substrate fluctuates significantly. As the thickness of the CVD diamond film increases with growth time, the SCD surface approaches the plasma, and the fluctuations in surface temperature increase. In other words, the internal stress increases, which increases the SCD basal plane bending.

## 4. Conclusions

To summarize, the substrate is the dominant factor for determining the basal plane bending in SCD. An effective method to alleviate basal plane bending is to use high-quality substrates with flat basal planes.

Growth temperature also strongly affects basal plane bending in SCD. A higher growth temperature increases the temperature gradient at the sample surface, which bends the basal plane. When the growth temperatures are 900 °C and 1150 °C, the radius of curvature of the lattice plane of CVD-grown diamond are 358.17 and 23.40 m, respectively. Increasing growth time also leads to more severe basal plane bending. For a growth time of 16 h, the radius of curvature of the lattice plane is 10.20 m.

Finally, increasing the temperature gradient at the sample surface also increases basal plane bending. To describe this phenomenon, we propose that the spherical distribution of the plasma and the distance between the plasma ball and the SCD surface are the main causes for basal plane bending in SCD.

## Figures and Tables

**Table 1:** Sample growth conditions on HTHP and MPCVD diamonds.

ID	Substrate	Growth Time (h)	Pressure (torr)	Methane/Hydrogen	Substrate Temperature (°C)
H1	HTHP diamond	4	275	3.00%	900
M1	MPCVD diamond	4	275	3.00%	900

**Table 2:** Sample growth conditions on HTHP diamonds at different temperatures.

ID	Substrate	Growth Time (h)	Pressure (torr)	Methane/Hydrogen	Substrate Temperature (°C)
H1	HTHP diamond	4	275	3.00%	900
H2	HTHP diamond	4	275	3.00%	1000
H3	HTHP diamond	4	275	3.00%	1100
H4	HTHP diamond	4	275	3.00%	1150

**Table 3:** Sample growth conditions on HTHP diamonds for different growth times.

ID	Substrate	Total Growth Time (h)	Pressure (torr)	Methane/Hydrogen	Substrate Temperature (°C)
H3	HTHP diamond	4	275	3.00%	1100
	HTHP diamond	8	275	3.00%	1100
	HTHP diamond	16	275	3.00%	1100
